# Effect of Maleic Anhydride-Modified Poly(lactic acid) on the Properties of Its Hybrid Fiber Biocomposites

**DOI:** 10.3390/polym9050165

**Published:** 2017-05-05

**Authors:** Abubakar Umar Birnin-Yauri, Nor Azowa Ibrahim, Norhazlin Zainuddin, Khalina Abdan, Yoon Yee Then, Buong Woei Chieng

**Affiliations:** 1 Department of Chemistry, Faculty of Science, Universiti Putra Malaysia, UPM Serdang, 43400 Selangor, Malaysia; norhazlin@upm.edu.my (N.Z.); ThenYoonYee@imu.edu.my (Y.Y.T.); chieng891@gmail.com (B.W.C.); 2 Department of Pure and Applied Chemistry, Kebbi State University of Science and Technology, P.M.B 1144, Aliero, 863102 Kebbi, Nigeria; 3 Materials Processing and Technology Laboratory, Institute of Advanced Technology, Universiti Putra Malaysia, UPM Serdang, 43400 Selangor, Malaysia; 4 Department of Biological and Agricultural Engineering, Faculty of Engineering, Universiti Putra Malaysia, UPM Serdang, 43400 Selangor, Malaysia; khalina@upm.edu.my; 5 Department of Pharmaceutical Chemistry, School of Pharmacy, International Medical University, No. 126, Jalan Jalil Perkasa 19, Bukit Jalil, 57000 Kuala Lumpur, Malaysia

**Keywords:** poly(lactic acid), maleic anhydride, hybrid biocomposites, bio-inspired

## Abstract

This work investigated the effect of maleic anhydride (MA)-modified poly(lactic acid) (PLA), which is melt-blended with different untreated and aqueous borax (BR)-treated hybrid oil palm empty fruit bunch fibers (EFBF)/Kenaf core fibers (KCF), and compression-molded into corresponding hybrid biocomposites. These hybrid systems includes BR-treated EFBF/BR-treated KCF reinforced MA-modified PLA i.e., BR(EFBF-KCF)-MAPLA, BR-treated EFBF/BR-treated KCF reinforced unmodified PLA i.e., BR(EFBF-KCF)-PLA, untreated EFBF/untreated KCF reinforced MA-modified PLA i.e., EFBF-KCF-MAPLA, and untreated EFBF/untreated KCF reinforced unmodified PLA i.e., EFBF-KCF-PLA respectively. Characterizations of the hybrid systems revealed that optimal mechanical, physical, morphological, thermal and dynamic mechanical properties were provided by the BR(EFBF-KCF)-MAPLA, resulting from improved interface adhesion, consequent of the synergistic influence of BR treatment of natural fibers, and the compatibilization effect provided by the MA-modified PLA. The grafting degree and efficiency of MA onto the PLA backbone was appreciable, as indicated by direct titration, and through monitoring using Fourier Transform Infrared Spectroscopy (FTIR); thus the MA-modified PLA facilitated the formation of strong interface adhesion with the BR-treated hybrid fibers. The BR(EFBF-KCF)-MAPLA showed promising properties for usage as a bio-inspired, and sustainable alternative fiberboard article.

## 1. Introduction

Polymer biocomposites have attracted growing research interest in relation to the utilization of naturally occurring lignocellulosic fibers and bio-based polymer matrices. Natural fiber is a broad term which refers to a wide range of plants, animals and mineral fibers [1]. Wood fibers and agro-based fibers such as bast and stem (e.g., hemp, jute, Kenaf etc.), leaf (e.g., banana, pineapple, sisal etc.), seed (oil palm, cotton etc.) are the commonly known fibers in the natural fiber based polymer composite industries [2].

Oil palm tree (*Eleis guineesis*) is a natural reservoir of biomass resource such as oil palm mesocarp fiber (OPMF), empty fruit bunch fiber (EFBF), oil palm trunk (OPT), oil palm fond (OPF), presses fruit fiber (PFF) etc. The oil palm tree is a major economic crop in southeast Asian countries such as Malaysia and Indonesia. It is also produced in large quantity in India and Latin America. The cultivation of oil palm plantation is commercially in large quantities, making the aforementioned countries the major producers in the world. Other oil palm producing countries include Columbia, Ecuador, Nigeria, Ivory Coast, Papua New Guinea and Thailand.The southeast Asian countries i.e., Malaysia and Indonesia, are the largest producers with ca. 85% of the world supply [3]. 

Malaysia is the world leading commercial exporter of palm oil and oil palm products having ca. 65% global market share. Oil palm plantations in Malaysia cover about six million hectares, and large quantities of biomass are generated approximately 100 million tons [3]. EFBF was reported to be the most abundant ca. 12.4 million tons per year [4]. EFBF is often unsystematically utilized by Malaysian oil palm industries as boiler fuel for energy generation, thereby contributing harmfully to atmospheric pollution. Despite that, an appreciable quantity is often left at the field as waste to decay. Leftover EFBF, upon decaying could contribute greatly to adverse environmental problems. Additionally, the Malaysian medium density fiberboard (MDF) mills use EFBF to supplement or substitute rubberwood as their basic raw material for production. The Agro-bio fiber Sdn Bhd Malaysia has patented EFBF-based MDF for over a decade [5]. The major issue worthy of concern here is sustaining the supply chain for EFBF raw materials, as there could be a likelihood that the demand may outstrip supply in the future, due to unsustainable applications. 

However, Malaysia has recently focused on the abundant production of Kenaf plant (*Hibiscus cannabinus L.*) for fiber production, largely to replace the cultivation and production of tobacco plants by farmers [6]. The Malaysian government has invested ca. RM 12 million on Kenaf research and subsequent development of the Kenaf based industry, through the National Kenaf Research and Development Program under the Ninth Malaysia Plan [7].

Kenaf was transported to Asia via the Mesopotamia territory by sea and caravan. India is the leading producer of the Kenaf plant. Other nations involved in the commercial production of Kenaf include Pakistan, China, Cambodia, Brazil, Cuba, Vietnam, Thailand, Indonesia and many countries in the African continents. Kenaf is rich in both bast and core fibers [8,9]. The Kenaf core fiber (KCF) is light-weighted and rich in lignin, though its high content of hemicellulose and low content of cellulose are its major drawbacks for biocomposite fabrication [10]. Therefore, hybridization of the EFBF and KCF may provide synergism in the material properties, due to possible compatibility between the two natural fibers. Also, hybridization could enable the sustainability of EFBF, considering the growth variation between the two fiber sources. The oil palm tree can take up to 2.5–4 years to grow [11,12], while Kenaf plants take only 150–60 days to attain maturity [13]. 

Previously, a positive hybrid effect in terms of the mechanical and physical properties of KCF incorporated-EFBF reinforced PLA biocomposites was reported by our research group [14]. The hybrid biocomposites developed in our previous work were intended to be used as an alternative to MDF, which popularly uses carcinogenic urea formaldehyde (UF) [15,16,17]. As a greener option, our previous work employed PLA as polymer matrix, owing to its attractive mechanical and environmental properties. PLA was previously reported to possess high strength, modulus and biocompatibility [18]. The hybrid biocomposites recorded higher density than the conventional MDF, which was resulted from high contents of hemicellulose and impurities in the EFBF and KCF used, as well as the brittle nature of the PLA. A major challenge associated with fabrication and utilization of the natural fiber reinforced biocomposite is the poor adhesion between the hydrophilic fiber and hydrophobic polymer [19,20]. This problem can be ascribed to the hydrophilic nature of the natural fibers and hydrophobicity of the polymer matrix [21]. Thus, the natural fibers need to be modified to produce an improved surface for bonding with the polymer matrices.

Several works were reported for the modification of natural fibers, involving physical, chemical and biological methods [22,23,24]. However, these reported methods suffered from some limitations regarding environmental and economic issues. Recently, borax (BR) was employed as a green chemical treatment technique, either as a filler or in its impregnated state [25,26,27]. Yet, BR in the aforesaid states, can unfavorably affect the material properties of biocomposites, perhaps, due to excess or unreacted BR in the biocomposites, which has hygroscopic properties [27]. Thus, an alternative method involving BR impregnation of EFBF and KCF, with a water-washing procedure was reported by us [28].

The BR-treated EFBF and KCF reported in our earlier work, significantly increased in cellulose and decreased in hemicellulose, with a partial reduction in lignin. Enhancements in the properties of the hybrid biocomposites was observed due to the BR treatment of the natural fibers. Yet, some anomalies were observed in the statistical differences among the tensile and flexural properties of the hybrid biocomposites, as well as some morphological defects, which were attributed to the brittle behavior of PLA, because the polymer was used in an unmodified state.

Unlike our preceding works, the novel approach in the present study intended to hybridize BR-treated EFBF as a primary fiber (55 wt %), and BR-treated KCF as a secondary fiber (5 wt %), and use these as reinforcements in maleic anhydride (MA)-modified PLA to form a BR(EFBF-KCF)-MAPLA hybrid system. Evaluation of the material performance of the BR(EFBF-KCF)-MAPLA hybrid system would be investigated and compared to those of its corresponding hybrid systems, such as BR-treated EFBF/BR-treated KCF reinforced unmodified PLA (i.e., BR(EFBF-KCF)-PLA), untreated EFBF/untreated KCF reinforced MA-modified PLA (i.e., EFBF-KCF-MAPLA), and untreated EFBF/untreated KCF reinforced unmodified PLA (i.e., EFBF-KCF-PLA). The integrated approaches of BR treatment of fibers, and compatibilization with MA-modified PLA were an attempt to follow the tricorner fabrication of hybrid biocomposites as previously posited by [29]. The tricorner design of hybrid fiber polymer biocomposites involved a chemical modification of the fiber reinforcements, as well as the polymer matrix, in order to achieve a relatively high performance in the synergistic properties of the hybrid biocomposites. 

To the best of our knowledge, this present work reports the first attempt to use MA-modified PLA for the compatibilization of BR-treated hybrid EFBF-KCF at a ratio of 55:5 weight percent loadings of the hybrid fiber reinforcements, aiming at synergistically improved material properties (i.e., mechanical, physical, morphological, thermal and dynamic mechanical) of the hybrid biocomposites.

## 2. Materials and Methods

### 2.1. Materials

Poly(lactic acid) was purchased from NatureWorks LLC (Minnetonka, MN, USA) under the trade name, polylactide resin 3052D. It has a melting point range between 170 and190 °C, a density ranging from 1.4 to 1.5 g·cm^−3^, and a molecular weight of 93,500 g/mol. Oil palm fiber, i.e., EFBF was obtained from Sabutek (M) Sdn. Bhd. (Kuala Lumpur, Malaysia). Kenaf core fiber, i.e., KCF was supplied by the Lembaga Kenaf dan Tembakau (Kelantan, Malaysia). Sodium tetraborate decahydrate (borax), maleic anhydride and all other chemicals were purchased from R & M Chemicals (Selangor, Malaysia).

### 2.2. Methods

#### 2.2.1. Fiber Purification

EFBF and KCF were purified by physical sorting, and then soaked in distilled water for 24 h at 25 °C, washed with warm water heated at 60 °C, cleaned with acetone (analytical grade, 99.5% purity), and placed in an oven to dry at 60 °C overnight. The oven-dried fibers were then ground using a commercial grinder (Waring products division, Torrington, CT, USA), sieved into sizes ranging from 300–400 µm, and then stored in sealed plastic bags at 25 °C for subsequent investigations.

#### 2.2.2. BR Treatment of Natural Fibers

Oven-dried, purified EFBF and KCF were chemically treated by soaking in an aqueous BR solution (5 wt % concentration), in accordance with our previous method [28]. The pH of the solution was measured to be alkaline at pH 9.1. The experimental temperature and time were 25 °C and 24 h, respectively. Afterward, the BR impregnated natural fibers were carefully washed with distilled water, with intermittent pH monitoring until a neutral pH was attained. Subsequently, the BR-treated natural fibers were oven-dried at 60 °C for 24 h. The oven-dried treated natural fibers were then kept in sealed plastic containers at room temperature for subsequent investigations.

#### 2.2.3. PLA Modification

The grafting of MA onto PLA was conducted via melt-blending in a Brabender Internal Mixer (Duisburg, Germany) at 170 °C, with a 50 rpm rotor speed for 15 min, in accordance with the method reported by [30], with some modifications. Initially, the PLA was oven-dried at 60 °C overnight in order to prevent its degradation due to hydrolysis during hot blending as previously posited by [18]. Oven-dried neat PLA (36.35 wt %), was melted in the Brabender for 5 min, benzoylperoxide (BPO) (0.75 wt %) was added and mixed for additional 2 min, and finally MA (3.00 wt %) was added and mixed continuously for 8 min. The blend was placed in a vacuum oven at 80 °C overnight to eliminate unreacted MA and BPO.

Purification was done by dissolving weighted MA grafted-PLA (2.00 g) in 40 mL chloroform, followed by addition of 0.75 mL of 1 M HCl in order to hydrolyze the anhydride into carboxylic acid at room temperature, in accordance with the reported method by [30]. Thereafter, the sample was precipitated by drop-wise addition into 400 mL acetone to remove the homo- copolymer of MA. The filtered precipitate was washed with acetone and distilled water several times, and vacuum oven-dried at 80 °C overnight. Thereafter, direct titration was employed to determine the degree of grafting. Purified PLA (0.4 g) was dissolved in 20 mL chloroform. The solution was titrated against sodium hydroxide (NaOH) in methanol (0.04 M) using a phenolphthalein indicator. The grafting degree was finally calculated using the volume and normality of NaOH (i.e., *N*_NaOH_ and *V*_NaOH_), as well as the sample weight (*m*_sample_) as shown in Equation (1).

(1) $$\mathrm{MA}\;\mathrm{grafting}\;(\%)=\frac{N_{\mathrm{NaOH}}V_{\mathrm{NaOH}}}{2000\times m_{\mathrm{sample}}}\times 98.06\times 100$$

Fourier transform infrared spectroscopy (FTIR) was employed to monitor the grafting process of MA onto the PLA backbone. The experiment was conducted using a Perkin Elmer Spectrum 100 series spectrophotometer (Waltham, MA, USA), which was equipped with an attenuated total reflectance (ATR) capacity. FTIR spectra were recorded at a wave number range of 400–4000 cm^−1^. The MA-modified PLA was examined, and comparison was made with neat PLA and pure MA respectively.

#### 2.2.4. Fabrication of Hybrid Fiber-PLA Biocomposites

To prepare the hybrid biocomposites, oven-dried BR-treated KCF (BRKCF) (5 wt %), as a secondary fiber, was mixed manually with BR-treated EFBF (BREFBF) (55 wt %), melt-blended with 40 wt % MA-modified PLA (MAPLA), and compression-molded into sheets with dimensions of 1 mm × 150 mm × 150 mm, and 3 mm × 150 mm × 150 mm (thickness × length × width). Compression molding was performed using a hydraulic hot press at 170 °C, 150 kg·cm^−12^, and 10 min molding temperature, pressure, and time, respectively. Cooling was then performed at 30 °C for 5 min. 

Corresponding hybrid biocomposites, using the same sample formulations as above, were similarly fabricated by melt-blending and compression-molding, these included untreated hybrid fiber (EFBF-KCF) reinforced unmodified PLA, untreated hybrid fiber (EFBF-KCF) reinforced MA-modified PLA, and BR-treated hybrid fiber (EFBF-KCF) reinforced unmodified PLA, respectively. The various sample formulations of the hybrid biocomposites are depicted in Table 1. 

## 3. Characterizations

### 3.1. Mechanical Properties

The tensile properties of the hybrid biocomposites were tested using a universal testing machine (Model 3365, Instron Corp., Norwood, MA, USA), equipped with a 5-kN load cell, 5 mm/min crosshead speed, and 25 °C experiment temperature. Five dog-bone shaped specimens were tested as specified by the ASTM D638-5 (2000) testing standard. Average values and standard deviation were reported for the tensile strength (TS), tensile modulus (TM), and elongation at break (EB) tests.

The flexural properties (three-point testing) of the hybrid biocomposites were performed using a universal testing machine (Model 3365, Instron Corp., Norwood, MA, USA), equipped with a 5-kN load cell, 1.3 mm/min crosshead speed, and 48 mm span length. The test was performed at 25 °C on five specimens with the dimensions 127.0 mm × 12.7 mm × 3.0 mm, in accordance with the ASTM D790 (2000) testing standard. Average values and standard deviation were reported for the flexural strength (FS) and flexural modulus (FM) tests.

The impact strength (IS) of the hybrid biocomposites was examined following the un-notched IZOD impact test, as specified by the ASTM D256 (2000) testing standard. The impact tester (Izod, Computerized, International Equipments, Mumbai, India) was equipped with a 7.5-J pendulum. The test was performed at 25 °C on five specimens with the dimensions, 63.5 mm × 12.7 mm × 3.0 mm, and the average values and standard deviations were reported.

### 3.2. Physical Properties

To test for the dimensional stability (i.e., water uptake and thickness swelling) of the hybrid biocomposites, test samples with the dimensions, 10.0 mm × 10.0 mm × 1.0 mm, were cut according to the ASTM D570 (2005) and EN 317 (1993) testing standards, respectively. The initial weight (*W*_1_) and thickness (*T*_1_) of the oven-dried test samples were measured and recorded. Test samples were then soaked in distilled water for 24 h at 25 °C. Thereafter, the samples were towel dried using tissue and measured for their second weight (*W*_2_) and thickness (*T*_2_). Five specimens for each sample were tested to determine the mean and standard deviation. The water uptake and thickness swelling were calculated using the following Equations (2) and (3) respectively:

(2) $$\mathrm{Water}\;\mathrm{Uptake}\;(\%)=\frac{W_{2}-W_{1}}{W_{1}}\times 100$$

(3) $$\mathrm{Thickness}\;\mathrm{Swelling}\;(\%)=\frac{T_{2}-T_{1}}{T_{1}}\times 100$$

Moreover, the densities of the hybrid biocomposites were measured in accordance with the BS EN 323 (1993) testing standard, as reported by the European Committee for Standardization [9]. The mass of the test samples having standard dimensions i.e., 1 mm × 150 mm × 150 mm, were obtained using an analytical balance. Afterwards, the volume of the test samples was determined using their dimensions, i.e., multiplying their length, width, and thickness, respectively. The density was subsequently calculated using Equation (4). Five tests were conducted, and average values and standard deviations were reported.


Density = Mass/Volume
(4)

### 3.3. Thermogravimetric Analysis

Thermogravimetric analysis (TGA) was employed to investigate the thermal stability of the hybrid biocomposites using the Perkin Elmer TGA7 Thermogravimetric analyzer (Waltham, MA, USA). Approximately 10 mg of each test samples were analyzed at 10 °C/min heating rate and 25 to 700 °C heating range, under a nitrogen atmosphere of 20 mL/min nitrogen flow rate. The sample weight losses were noted and plotted against the heating temperatures. 

### 3.4. Dynamic Mechanical Analysis

The stiffness and damping (i.e., modulus and tan delta) of the hybrid biocomposites were measured using the Dynamic Mechanical Analyzer (DMAQ800, TA instruments, Newcastle, DE, USA) by means of bending mode according to ASTM D5023-01, as previously reported by [31]. The test samples, having a dimension of 63.5 mm × 12.7 mm × 3.0 mm (length × width × thickness) were analyzed at a temperature scan range of 25 to 170 °C, heating rate of 2 °C/min and 1 Hz frequency of dynamic force under nitrogen atmosphere. The stiffness or modulus i.e., storage modulus (E′) and loss modulus (E′′), as well as damping or tan delta i.e., loss factor (tan *δ*) of each sample were recorded and plotted as function of temperature. 

### 3.5. Morphological Analysis of Fractured Surfaces

Scanning electron microscopy (SEM) was employed to analyze the morphologies of the fractured surfaces of the hybrid biocomposites. The experiment was conducted using a LEO 1455 VP scanning electron microscope (Zeiss, Jena, Germany) operated at a 10 kV accelerating voltage. The oven-dried samples were held in place using a metal holder of the instrument. Afterward, conductivity of the samples was enhanced by gold coating for 3 min using a Bio-Rad^TM^ coating system (Hercules, CA, USA) before the commencement of the experiment.

## 4. Results and Discussion

The influence of MA-modified PLA on the mechanical, physical, thermal, dynamic mechanical and morphological properties of its hybrid biocomposites reinforced with BR-treated EFBF/KCF were investigated, and the test results are presented and discussed in the succeeding subsections.

### 4.1. Grafting Degree, Efficiency and FTIR Spectra of MA-Modified PLA

**Figure 1:**
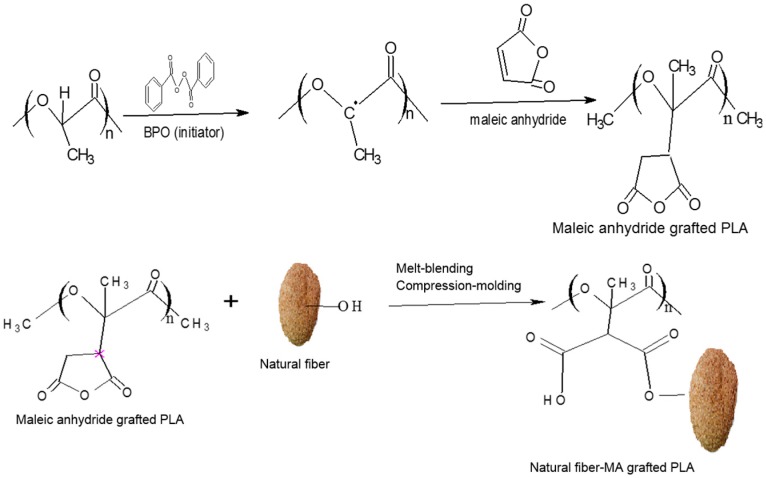
Hypothetical mechanism for MA grafting of PLA, and compatibilization with fibers.

There are three major steps involved in the mechanism for the MA grafting of PLA, and its subsequent compatibilization to natural fibers (Figure 1). Firstly, the PLA reacts with BPO (initiator), where deprotonation occur and PLA macroradicals (PLA*) are generated. Secondly, the MA reacts with the PLA macroradicals to form MA-grafted PLA, which lastly, form bonds with the natural fibers, and provides compatibilization effects.

The degree and efficiency of MA grafting onto the PLA backbone was probed by direct titrimetric method, and calculated to be 0.87% and 16.58%, respectively. Both the grafting degree and efficiency was low, which may be have been a result of PLA degradation that occurred, consequent of thermal decomposition of BPO and MA on the PLA backbone during melt-blending.

Several works previously reported that extrusion and reactive melt-blending often cause low grafting efficiency [32,33]. Nonetheless, the degree of grafting observed, appeared to be slightly higher than the one previously reported by [34], where the authors used relatively higher molecular weight (*M*_w_) PLA. In this study, the low degree of MA grafting onto PLA could be associated with the low *M*_w_ of the PLA used. The *M*_w_ of PLA is 93,500 g/mol, which is previously reported to be a low *M*_w_ PLA by [35]. Low *M*_w_ PLA often provides a higher grafting percentage than the high *M*_w_ PLA. The PLA molecules suffers entanglement during the MA grafting reaction of high *M*_w_ PLA, thereby restricting efficient diffusion of reacting molecules onto the PLA backbone, hence, hampering the grafting efficiency. On the other hand, the results for the MA grafting onto PLA appeared to be lower than the observation reported by [32], where the authors enhanced MA grafting onto PLA using a styrene comonomer.

**Figure 2:**
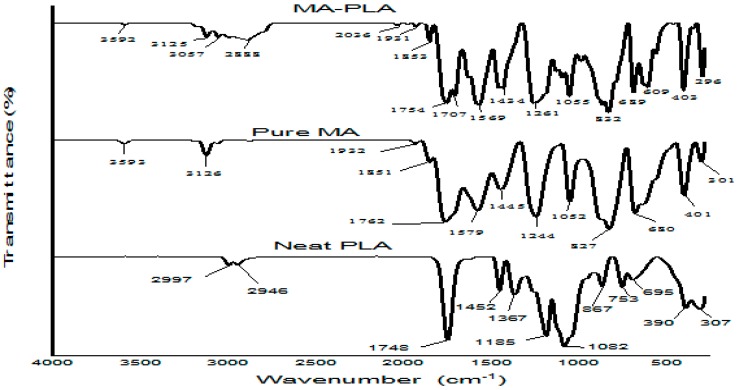
FTIR spectra of neat PLA, Pure MA and MAPLA.

The grafting reaction of MA onto PLA backbone was monitored by FTIR analysis. FTIR was performed on MA-grafted PLA samples which were previously purified by eliminating residual (unreacted) MA. The FTIR spectra are depicted in Figure 2. The absorption band at 3593 cm^−1^ in the spectra of pure MA can be assigned to the symmetric OH stretching vibration of water partially hydrogen-bonded with the hydrophilic carbonyl group of MA [36]. A similar peak appeared at 3592 cm^−1^ in the spectra of MA-PLA, but did not appear in the neat PLA spectra. The =C–H stretching vibration band of anhydride was located at 3126 cm^−1^ in pure MA, and at 3125–3057 cm^−1^ in MA-PLA, but disappeared in the neat PLA. This implies that the MA was grafted onto the PLA.

The absorption band at 2997 cm^−1^ in neat PLA, was assigned to the C–H stretching of the methyl groups of PLA, this band decreased to 2888 cm^−1^ in the MAPLA spectra, and did not appear in the pure MA spectrum. The peak at 1748 cm^−1^ in neat PLA, corresponded to –C=O stretching of the carbonyl group in PLA, which increased to 1754 cm^−1^ in the spectra of MAPLA, perhaps due to grafting reaction. The presence of MA in the FTIR spectra of MA-grafted PLA was previously described by the qualitative appearance of two typical –C=O stretching modes at 1756 and 1850 cm^−1^ for intense symmetric and weak asymmetric vibrations, respectively [37,38]. In the present work, these peaks appeared at 1754 and 1853 cm^−1^, perhaps, due to the strong intensity of the neighboring –C=O stretching of PLA. This observation confirmed the low grafting performance of the melt-blending technique as previously posited by [33].

The peaks at 1452 and 1367 cm^−1^ in PLA, were ascribed to the –C–H deformation and C–O–H absorption bands, respectively. These peaks reduced to 1434 and 1261 cm^−1^ in MAPLA, perhaps due to the grafting reaction. The reduction of 1185 cm^−1^ of PLA to 1055 cm^−1^ in MAPLA, indicated –C–O– stretching of the anhydride group from MA [39]. The absorption peaks at 867 cm^−1^ in PLA decreased to 832 cm^−1^ in MAPLA. These peaks were associated with =C–O–C= asymmetric ring-stretching vibration of the saturated cyclic five-membered anhydride compound, as previously observed by [40]. Lastly, the bands at 753 cm^−1^ in PLA declined to 689 cm^−1^ in MAPLA, and were assigned as C–H bending vibrations of the anhydride ring involved in the grafting reaction. Generally, the FTIR spectra indicated that the grafting reaction between the PLA and MA successfully occurred.

### 4.2. Mechanical Properties

The mechanical properties i.e., TS, EB, TM, FS, FM, and IS of all the hybrid biocomposites are given in Table 2. The TS of EFBF-KCF-PLA hybrid system recorded 35.59 MPa, but declined in EFBF-KCF-MAPLA by about 15.10%. Approximately 5.19% of increase was recorded by BR(EFBF-KCF)-PLA compared to EFBF-KCF-PLA. These disparities in TS may be attributed to poor fiber-to-PLA interface bonding, arising due to the hydrophilic nature of the fibers and the hydrophobicity of the polymer matrix [41].

The inclusion of untreated hybrid fibers into MA-modified PLA (i.e., EFBF-KCF-MAPLA), provided a TS slightly lower to those involving BR-treated hybrid fibers reinforced unmodified PLA (i.e., BR(EFBF-KCF)-PLA), having an insignificant difference. Upon combining the two methods via the tricorner approach i.e., BR(EFBF-KCF)-MAPLA, a synergistically improved TS was obtained, presenting an approximately 33.36%, 53.75% and 26.98% increase in TS, compared to the EFBF-KCF-PLA, EFBF-KCF-MAPLA, and BR(EFBF-KCF)-PLA hybrid systems respectively. This could be due to BR elimination of hemicellulose and impurities, and increased cellulose content which enabled the fibers to form strong interface adhesions with the MA-modified PLA, through reactive compatibilization.

The EB of the EFBF-KCF-PLA hybrid system recorded approximately 93.36% lower compared to the BR(EFBF-KCF)-MAPLA. EFBF-KCF-MAPLA and BR(EFBF-KCF)-PLA hybrid systems presented almost similar EBs, which were higher than EFBF-KCF-PLA, but lower than BR(EFBF-KCF)-MAPLA hybrid systems. This implies that inclusion of the BR-treated hybrid fibers into the MA-modified PLA provided the best EB, which could be due to synergistically improved interface bonds.

The TM of the BR(EFBF-KCF)-MAPLA showed a slight reduction compared to the EFBF-KCF-PLA, perhaps due to the higher fiber-fiber interaction occurring due to the weakened interface bond in the EFBF-KCF-PLA hybrid system, thereby making the high modulus of the natural fibers dominant in the hybrid biocomposite. The reduced fiber-fiber interactions consequent of the strengthened interface bond, and molecular rearrangements caused by the MA in the BR(EFBF-KCF)-MAPLA tended to slightly decrease the TM. A similar trend can be seen between the EFBF-KCF-MAPLA and BR(EFBF-KCF)-PLA hybrid systems.

The EFBF-KCF-PLA hybrid system presented a FS of 25.08 MPa, which recorded a percentage increase of approximately 88.72%, 60.53%, and 119.14% for EFBF-KCF-MAPLA, BR(EFBF-KCF)-PLA, and BR(EFBF-KCF)-MAPLA respectively. Clearly, the BR(EFBF-KCF)-MAPLA hybrid systems recorded the highest increase in FS, which can be imputed to the synergistically improved interface bond, resulted due to BR treatment of fibers and MA modification of PLA.

The BR(EFBF-KCF)-PLA hybrid system recorded the highest FM, while BR(EFBF-KCF)-MAPLA presented the lowest. The EFBF-KCF-PLA and EFBF-KCF-MAPLA hybrid systems presented almost similar FMs. These disparities observed in the FM of the hybrid systems may be associated with the short lengths of the natural fibers used, which also tended to unevenly be distributed or dispersed in the hybrid biocomposites.

The IS of the EFBF-KCF-PLA recorded 12.29 J/m, which was a percentage increase of approximately 31.16%, 36.29%, and 164.69% for EFBF-KCF-MAPLA, BR(EFBF-KCF)-PLA, and BR(EFBF-KCF)-MAPLA respectively. Clearly, BR(EFBF-KCF)-MAPLA presented the highest increase, which implies that the combined methods of BR treatment of fibers and MA modification of PLA produces stronger hybrid biocomposites, which can absorb high impact energy without crack generation.

Generally, the best mechanical properties were provided by the BR-treated hybrid fiber reinforced MA-modified PLA i.e., BR(EFBF-KCF)-MAPLA hybrid system, which is an indication of synergistically improved interface bonds provided by the BR-treated natural fibers and MA-modified PLA.

### 4.3. Physical Properties

The physical properties i.e., dimensional stability, which includes water uptake (WU) and thickness swelling (TSW), as well as the density of all the hybrid biocomposites were examined and the test results are presented in Table 3.

It was seen that the smallest WU, TSW, and density was present in BR(EFBF-KCF)-MAPLA. This may have been due to BR elimination of hydrophilic hemicellulose and impurities from the fibers, coupled with MA modification of PLA, which strengthened the interface bond, thereby making the hybrid system impassable by moisture, as well as other foreign bodies. The presence of these hemicelluloses and impurities in the EFBF-KCF-PLA and EFBF-KCF-MAPLA, may have contributed to their relatively higher WU, TSW, and density.

Similarly, the lowest density provided by the BR(EFBF-KCF)-MAPLA hybrid system (Table 3), could be explained based by its better interface adhesion, with the product consequently possessing no voids, gaps, or micro-cracks. When such defects are present in the hybrid biocomposites, they may serve as penetration points for foreign agents such as water and air, into the hybrid biocomposite, thereby adding to its density. The presence of these voids, gaps and micro-cracks in the EFBF-KCF-PLA, EFBF-KCF-MAPLA, and BR(EFBF-KCF)-PLA hybrid systems, as would be visible in their morphologies, and may have contributed to their somewhat higher densities.

The lowering of density was an improvement achieved due to MA modification of PLA, as earlier asserted. Yet, the density for the BR(EFBF-KCF)-MAPLA hybrid system is similar to high density fiberboard (HDF), because it is higher than the density range of 495–801 kg·m^−3^ previously reported for conventional MDF, by [42]. 

Generally, a good correlation was established between the mechanical and physical properties of the hybrid biocomposites; in both, the best results were presented by the BR(EFBF-KCF)-MAPLA hybrid system, consequent of the synergistic improvement of the interface bond, obtained as a result of the combined approaches of BR treatment of natural fibers and MA modification of PLA.

### 4.4. Statistical Analysis

One-way analysis of variance (ANOVA) was used to test the significant difference regarding the mechanical properties (Table 4) and physical properties (Table 5) of the hybrid biocomposites.

The variance of the mechanical properties (i.e., TS, EB, TM, FS, FM, and IS) and physical properties (i.e., WU, TSW and Density) of the samples was decomposed into two components by ANOVA analysis viz., the sum of square between group (SSBG) and the sum of square within group (SSWG). The F-ratio was calculated as a ratio of the SSBG estimate to the SSWG estimate.

It can clearly be seen from Table 4 and Table 5 that the p value of the *F*-test for both mechanical and physical properties was less than 0.05, which indicates that there was a statistically significant difference between the mean mechanical and physical properties from untreated hybrid fiber-unmodified PLA, untreated hybrid fiber-MA modified PLA, BR-treated hybrid fiber-unmodified PLA to their corresponding BR-treated hybrid fiber-MA modified PLA at 95.0% confidence level.

Generally, the tricorner approach in the fabrication of the hybrid biocomposites by BR treatment of natural fibers and compatibilization with MA modified PLA, provided a synergistically improved mechanical and physical properties of the hybrid biocomposites, having statistical significance.

### 4.5. Thermogravimetric Analysis

**Figure 3:**
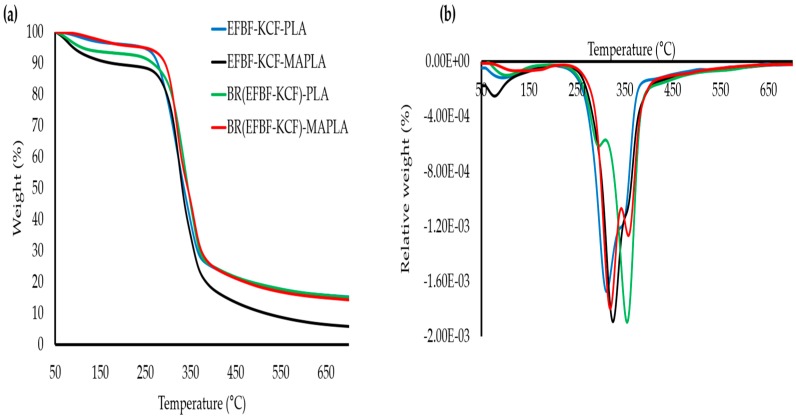
Thermogravimetric analysis (TGA) (**a**), and DTG (**b**) thermograms of hybrid biocomposites.

TGA was employed to probe the thermal stability of the hybrid biocomposites by measuring their degree and rate of decomposition or change in mass, as a function of temperature in a controlled atmosphere. The TGA thermograms for the hybrid biocomposites are presented in Figure 3a. 

From Figure 3a, clearly, the untreated EFBF-KCF-PLA showed a sharper thermogram around 50–160 °C, which can be assigned to the moisture evaporation processes, and the loss of volatile products. The hybrid system EFBF-KCF-PLA, contained a high amount of hemicellulose and impurities, hence, its moisture content should have been higher. The initial and maximum decomposition temperatures (i.e., *T*_onset_ and *T*_max_) of the EFBF-KCF-PLA tended to rise slightly after the fibers were treated with aqueous BR solution i.e., BR(EFBF-KCF)-PLA (Figure 3a). This indicates that the elimination of hemicellulose along with other fiber surface impurities by the BR, improved the thermal resistance of the hybrid biocomposites [43,44].

Synergistically improved thermal stability was provided by BR(EFBF-KCF)-MAPLA, which may have been a result of the strong interface bonds formed by the BR-treated hybrid fiber and MA-modified PLA. The rich-lignin content of KCF could also aid the thermal stability of the hybrid biocomposites. These results correlated with earlier findings on the mechanical and physical properties of the hybrid biocomposites.

However, the DTG thermograms for the hybrid biocomposites given in Figure 3b, shows that, beside the weight losses around 50–160 °C which can be ascribed to moisture evaporation processes, two additional thermograms also appeared. The first thermogram appeared at around 250–300 °C, and was assigned as hemicellulose and early cellulose decomposition. Hemicellulose degrades at higher rate because it is more thermally unstable than cellulose and lignin [45].

The second thermogram appeared at around 300–450 °C, and was ascribed as late cellulose and early lignin decomposition. Lignin decomposes slowly and covers a wide temperature range, owing to its different oxygen-containing functional groups which possess varying thermal stabilities [46,47]. The residue ash recorded at 700 °C includes EFBF-KCF-PLA (10.23%), EFBF-KCF-MAPLA (14.99%), BR(EFBF-KCF)-PLA (18.06%), and BR(EFBF-KCF)-MAPLA (17.44%). Generally, increase in thermal degradation temperature and residue char formation was observed due to BR treatment of the natural fibers and MA modification of PLA. This observation agreed with previous literature [48,49].

### 4.6. Dynamic Mechanical Analysis (DMA)

**Figure 4:**
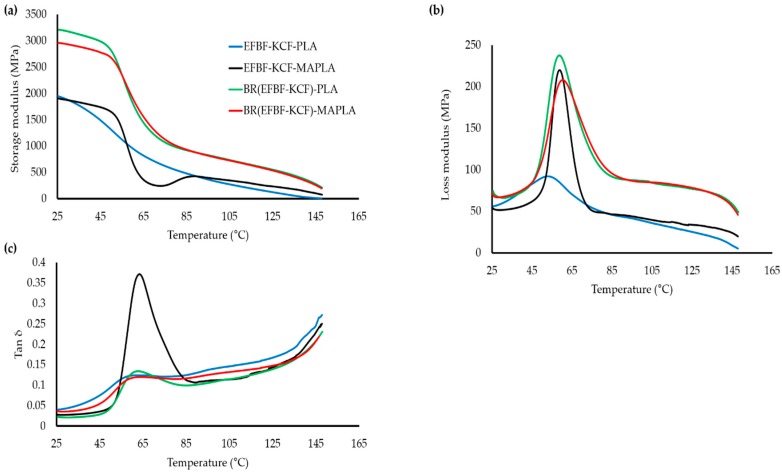
Storage modulus (**a**); loss modulus (**b**); and tan*δ* (**c**) curves of hybrid biocomposites.

The results on dynamic mechanical properties which depict the temperature dependence on dynamic storage modulus (Figure 4a), loss modulus (Figure 4b) and tan*δ* (Figure 4c) for the hybrid biocomposites are presented. From Figure 4a, the storage modulus value at 25 °C for EFBF-KCF-PLA hybrid system was 1936 MPa, which increased upon BR treatment of fibers i.e., BR(EFBF-KCF)-PLA (1952 MPa). Further significant improvement was noted in the hybrid system, involving BR-treated hybrid fiber reinforced MA modified PLA i.e., BR(EFBF-KCF)-MAPLA (2998 MPa). This trend remained similar even at higher temperatures, hence suggesting that BR(EFBF-KCF)-MAPLA has a better storage modulus among the hybrid systems. Previously, addition of MA has been reported to improve the storage modulus of PP-clay hybrid [50]. Also, the storage modulus of all the hybrid systems tended to decrease upon temperature rise, perhaps due to the change in the nature of the materials from plastic to viscous [51,52,53].

The EFBF-KCF-PLA hybrid system showed a minimal value of loss modulus (Figure 4b) at 65 °C. Similar minimal loss modulus values for natural fiber thermoplastic composites were also observed by [54]. However, hybridization of KCF with EFBF, improved the loss modulus at higher temperatures, which could be due to enhanced fiber-polymer interfacial bonding, occurring due to the reinforcing ability of lignin-rich KCF. The *T*_g_ values recorded included EFBF-KCF-PLA (52.90 °C), EFBF-KCF-MAPLA (58.86 °C), BR(EFBF-KCF)-PLA (58.68 °C), and BR(EFBF-KCF)-MAPLA (60.32 °C). Thus, in terms of *T*_g_, BR(EFBF-KCF)-MAPLA possessed optimal thermal stability, consequent of the synergistic enhancement in the interface adhesion, provided by the BR-treated hybrid fibers and MA-modified PLA. This observation interrelated with earlier statements on the TGA, and the mechanical and physical properties of the hybrid biocomposites.

Figure 4c shows the tan δ peaks of the hybrid biocomposites, which clearly showed that, at 25 °C, the lowest results were attained by BR(EFBF-KCF)-MAPLA compared to BR(EFBF-KCF)-PLA, EFBF-KCF-MAPLA and EFBF-KCF-PLA, respectively. This showed that hybrid systems involving the combined methods of BR treatment of fiber and compatibilization with MA-modified PLA provided lowest tendency to extricate energy, which suggests that they have a better thermal stability in relation to those hybrid systems produced using separate modification methods.

### 4.7. Morphology of Fractured Surfaces

**Figure 5:**
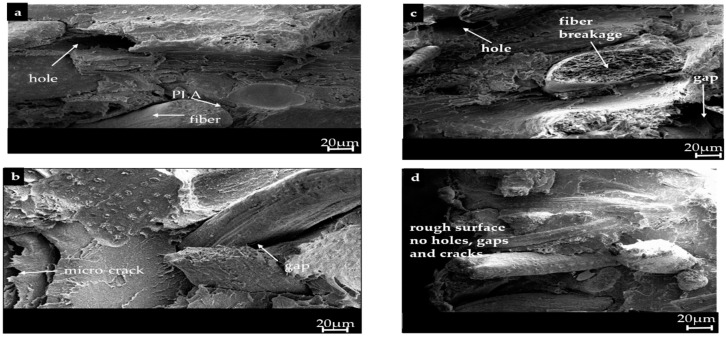
Morphology of fractured surfaces of (**a**) EFBF-KCF-PLA; (**b**) BR(EFBF-KCF)-PLA; (**c**) EFBF-KCF-MAPLA; and (**d**) BR(EFBF-KCF)-MAPLA.

The morphologies of the fractured surfaces of the hybrid systems i.e., EFBF-KCF-PLA (Figure 5a), EFBF-KCF-MAPLA (Figure 5b), BR(EFBF-KCF)-PLA (Figure 5c), and BR(EFBF-KCF)-MAPLA (Figure 5d) are shown respectively. The hybrid system i.e., BR(EFBF-KCF)-MAPLA showed rough surfaces with no holes, micro-cracks or gaps (Figure 5d), perhaps due to better interface adhesion provided by the BR-treated hybrid fibers, and compatibilization effect provided by the MA-modified PLA. This finding agreed with earlier results on the DMA, TGA, mechanical and physical properties stated. In the micrograph of BR(EFBF-KCF)-PLA (Figure 5c), few cracks and gaps were seen. Those of EFBF-KCF-MAPLA (Figure 5b) and EFBF-KCF-PLA (Figure 5a) showed holes, micro-cracks and gaps, which may have contributed to their relatively lower material properties (i.e., mechanical, physical, thermal and dynamic mechanical), arising due to weaker interface adhesion from a high content of hemicellulose, and the brittle behavior of the unmodified PLA [55], especially in the hybrid system EFBF-KCF-PLA.

## 5. Conclusions

This study demonstrated that MA modification of PLA could aid in the synergistic improvement of the mechanical, physical, thermal, dynamic mechanical and morphological properties of hybrid biocomposites fabricated using a combination of BR-treated EFBF and KCF as reinforcements i.e., BR(EFBF-KCF)-MAPLA. This hybrid system, compared to its corresponding systems, was observed to possess relatively better interface adhesion, which consequently enhanced its tensile, flexural, impact, thermal and dynamic mechanical properties. The water uptake and thickness swelling of the BR(EFBF-KCF)-MAPLA hybrid system was less pronounced, and its density was lower compared to its corresponding hybrid systems. However, its recorded density was higher than those of conventional MDF, and similar to the density of HDF. The application of the BR(EFBF-KCF)-MAPLA hybrid biocomposites, compared to its corresponding ones, as alternative fiberboards is potentially promising, as a cleaner and sustainable option to conventional fiberboard usage.

## Figures and Tables

**Table 1:** Hybrid fiber-poly(lactic acid) (PLA) mixing formulations.

Sample Code	EFBF (wt %)	KCF (wt %)	PLA (wt %)	BR Modifier	MA (wt %)	BPO (wt %)
EFBF-KCF-PLA	55	5	40	-	-	-
EFBF-KCF-MAPLA	55	5	36.25	-	3	0.75
BR(EFBF-KCF)-PLA	55	5	40	yes	-	-
BR(EFBF-KCF)-MAPLA	55	5	36.25	yes	3	0.75

EFBF: Empty fruit bunch fibers, KCF: Kenaf core fibers, MA: maleic anhydride, MAPLA: MA-modified PLA, BR: Borax-treated.

**Table 2:** Mechanical properties of hybrid biocomposites.

Sample	TS (MPa)	EB (%)		TM (MPa)	FS (MPa)	FM (GPa)	IS (J/m)
EFBF-KCF-PLA	35.59 ± 1.32	8.59 ± 0.84	469.39 ± 24.11		25.08 ± 1.80	4.19 ± 0.20	12.29 ± 0.50
EFBF-KCF-MAPLA	30.92 ± 2.82	12.62 ± 1.29	248.64 ± 39.05		47.33 ± 3.34	4.65 ± 0.43	16.12 ± 0.49
BR(EFBF-KCF)-PLA	37.44 ± 2.56	12.23 ± 0.45	305.92 ± 20.64		40.26 ± 1.19	5.23 ± 0.15	16.75 ± 0.49
BR(EFBF-KCF)-MAPLA	47.54 ± 14.50	16.61 ± 4.09	373.94 ± 14.29		54.96 ± 6.53	2.49 ± 0.10	32.53 ± 2.18

TS: Tensile strength, EB: Elongation at break, TM: Tensile modulus, FS: Flexural strength, FM: Flexural modulus, IS: Impact strength. ± refers to standard deviation.

**Table 3:** Physical properties of hybrid biocomposites.

Sample	WU (%)	TSW (%)	Density (kg·m^−3^)
EFBF-KCF-PLA	8.50 ± 0.54	4.15 ± 0.75	1066 ± 16.85
EFBF-KCF-MAPLA	10.17 ± 0.58	5.47 ± 0.61	1072 ± 16.33
BR(EFBF-KCF)-PLA	6.30 ± 0.69	3.12 ± 0.30	992 ± 27.90
BR(EFBF-KCF)-MAPLA	4.25 ± 0.35	2.50 ± 0.25	932 ± 26.12

WU: Water uptake, TSW: Thickness swelling. ± refers to standard deviation.

**Table 4:** ANOVA Test for mechanical properties of hybrid biocomposites.

Source	TS	EB	TM	FS	FM	IS
TSS	1599.419042	210.7796105	307481.1619	3057.850788	28.69395115	1225.5417
SSWG	961.7113	67.058795	172898.8654	953.845995	9.1344369272	21.99148
SSBG	637.7077421	143.7208155	134582.2965	2104.004793	19.55951422	1203.55022
DFBG	3	3	3	3	3	3
DFWG	16	16	16	16	16	16
*F*-ratio	3.53651658	5.333333333	4.151399412	11.76432984	11.42023422	291.882787
*p*-value	0.038846	0.009711	0.023566	0.000254	0.000298	0.00001

ANOVA: analysis of variance; TS: tensile strength; EB: elongation at break; TM: tensile modulus; FS: flexural strength; FM: flexural modulus; IS: impact strength; TSS: total sum of square; SSWG: sum of square within group; SSBG: sum of square between group; DFBG: degree of freedom between group; DFWG: degree of freedom within group; F: F test for ANOVA, P-value: probability value; Number of sample = 4; Number of observations = 20.

**Table 5:** ANOVA Test for physical properties of hybrid biocomposites.

Source	WU	TSW	DT
TSS	104.804895	29.683095	72,303.75
SSWG	4.8874	4.35928	6640
SSBG	99.917495	25.323815	65,663.75
DFBG	3	3	3
DFWG	16	16	16
*F*-ratio	109.0341095	30.98226007	52.74197
*p*-value	0.00001	0.00001	0.00001

ANOVA: analysis of variance; WU: water uptake; TSW: thickness swelling; DT: density; TSS: total sum of square; SSWG: sum of square within group; SSBG: sum of square between group; DFBG: degree of freedom between group; DFWG: degree of freedom within group; F: *F* test for ANOVA, *p*-value: probability value; Number of sample = 4; Number of observations = 20.
